# Acute coronary syndrome: a rare case of multiple endocrine neoplasia syndromes with pheochromocytoma and medullary thyroid carcinoma

**DOI:** 10.7497/j.issn.2095-3941.2015.0016

**Published:** 2015-09

**Authors:** Alessadro Maloberti, Paolo Meani, Roberto Pirola, Marisa Varrenti, Marco Boniardi, Anna Maria De Biase, Paola Vallerio, Edgardo Bonacina, Giuseppe Mancia, Paola Loli, Cristina Giannattasio

**Affiliations:** ^1^Health Science Department, Milano-Bicocca University, Milan 20159, Italy; ^2^Cardiology IV, “A. De Gasperis” Department, Ospedale Niguarda Ca’ Granda, Milan 20159, Italy; ^3^General Oncologic and Mini-invasive Surgery Department, ^4^Anatomy and Histology Department, ^5^Endocrine Unit, Niguarda Ca’ Granda Hospital, Milan 20159, Italy

**Keywords:** Secondary hypertension, pheochromocytoma, echocardiography, medullary thyroid carcinoma, multiple endocrine neoplasia

## Abstract

Pheochromocytoma is a tumor arising from neuroectodermal chromaffin tissues in the adrenal gland or extra-adrenal paraganglia (paragangliomas). The prevalence of the tumor is 0.1%-0.6% in the hypertensive population, of which 10%-20% are malignant. Pheochromocytoma produces, stores, and secretes catecholamines, as well as leads to hypertensive crisis, arrhythmia, angina, and acute myocardial infarction without coronary artery diseases. We report a case of acute coronary syndrome (ACS) with a final diagnosis of multiple endocrine neoplasia with pheochromocytoma and medullary thyroid carcinoma (MTC).

## Introduction

Pheochromocytoma, a tumor arising from neuroectodermal chromaffin tissues, produces, stores, and secretes catecholamines^1^. The prevalence of the tumor is approximately 0.1%-0.6% in the hypertensive population^2^, of which the majority of the cases arise from adrenal medulla and 10%-20% from extra-adrenal paraganglia (paragangliomas)^3^. Approximately 10%-20% of pheochromocytomas are malignant^4^, and genetic factors are implicated in 10%-20% of the cases, particularly in Von Hippel–Lindau, multiple endocrine neoplasia type 2, and neurofibromatosis^5^^,^^6^.

Typical presentations of pheochromocytoma include episodic headache, sweating, and tachycardia. Several patients with pheochromocytoma manifest paroxysmal hypertension and hypertensive crisis with sustained hypertension or normal blood pressure (BP)^1^. Other patients present cardiovascular events, including cardiac arrhythmias (sinus tachycardia or bradycardia and supraventricular arrhythmias), heart failure by toxic cardiomyopathy, angina, and acute coronary syndrome (ACS), in the absence of coronary artery diseases^7^. Myocardial ischemia is mediated by adrenergic mechanisms, including catecholamine induction of myocardial oxygen demand and coronary vasospasm. Patients with pheochromocytoma present a 14-fold higher risk of cardiovascular events than that of hypertensive cases^8^^,^^9^.

Although several cases and retrospective analyses described the diagnosis of pheochromocytoma after ACS presentation^10^^-^^20^, only a few studies reported this condition in the setting of hyperthyroidism and/or genetics^21^^,^^22^.

## Case report

A 49-year-old Asian man with oppressive chest pain associated with hypertensive crisis (BP, 200/100 mmHg), with no other symptoms, was referred to our hospital. The patient’s medical history included hypertension and smoking. Sixteen years prior to the current condition, the patient had undergone surgical mitral and aortic valve substitution with mechanical prosthesis and followed by re-substitution because of secondary thrombosis. He had a history of paroxysmal atrial fibrillation, which was effectively controlled using 200 mg of amiodarone. However, the medication was stopped 2 weeks before the admission because of evidence of thyrotoxicosis [thyroid-stimulating hormone, 0.008 µIU/mL; free triiodothyronine (fT3), 8.6 pg/mL; and thyroxine (fT4), 43.0 pg/mL], which was treated with steroid, potassium perchlorate, and methimazole.

Upon admission, the patient was oriented and alert, with a BP of 200/100 mmHg, heart rate of 100 bpm, and normal physical examination results. The electrocardiogram showed sinus tachycardia and non-specific ST-segment and T-wave changes. Laboratory examination results showed increased levels of myocardial necrosis enzymes (troponine T-hs, 133 ng/L; and creatine kinase−MB isoenzyme, 13.0 ng/L) and thyroid hormones (TSH, 0.007 µIU/mL; fT3, 8.3 pg/mL; and fT4, 44.2 pg/mL).

After the patient was initially diagnosed with ACS, echoscope was performed and showed a modest and diffuse decrease of left ventricular contraction with a conserved ejection fraction (0.50) but color Doppler analysis indicated normal heart valves. Coronary angiography did not show any clinically relevant coronary lesion, whereas thyroid echography demonstrated a heterogeneous structure in the absence of cysts and nodules. Without evidence of coronary diseases but with initial response to thyreostatic and steroid administration (fT3, 5.4 pg/mL; and fT4 36.2 pg/mL), the patient was finally diagnosed with ACS caused by hyperthyroidism. The patient was discharged and followed up by an endocrinologist and a cardiologist.

After 5 months, the patient was hospitalized again because of chest pain associated with hypertensive crisis (BP, 280/120 mmHg), nausea, headache, and vomiting. He was unresponsive to therapy with oral beta-blockers, alpha-blockers, and clonidine.

As the patient’s clinical case history was highly suggestive of pheochromocytoma, bioassays were performed. The following results were obtained: 24-h urinary epinephrine, 547 μg/24 h; 24-h urinary 633 μg/24 h; 24-h urinary metanephrine, 8,720 μg/24 h; 24-h urinary, 4,120 μg/24 h; serum calcitonin, 312 pg/mL; and normal parathormon, 34 pg/mL.

**Figure 1:**
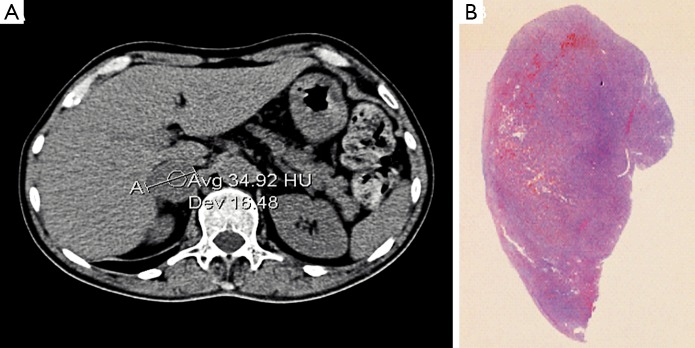
(A) Pheochromocytoma: abdominal computed tomography showed a 35 mm right adrenal lesion with smooth margins and a density higher than 2 HU. (B) Histological findings, H&E staining.

A subsequent whole-body computed tomography (CT) showed a 35 mm right adrenal lesion (**Figure 1****,** **Figure 1A**) with smooth margins and a density higher than 2 HU. The diagnosis was right adrenal pheochromocytoma with suspected medullary thyroid carcinoma (MTC) with the MEN2A syndrome. After collegial discussion, the patient was treated with right adrenalectomy and thyroidectomy, as well as central compartment lymphadenectomy and parathyroid exploration.

**Figure 2:**
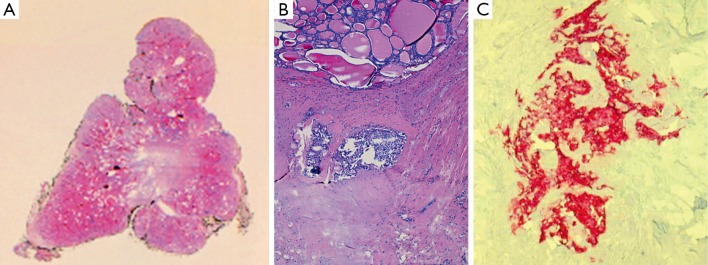
Medullary thyroid carcinoma: histological findings. (A) Scan of original histological slide (H&E staining, pale central area corresponded to dense fibrous tissue infiltrated by tumor). (B) Two tumor nodules in dense fibrous tissue at the top of figure (H&E staining, 100×). (C) Tumor cells are positive for Calcitonin (Calcitonin staining, 200×).

Macroscopic examination indicated normal results. The thyroid sample showed intra-parenchymal isolation of the right lobe, with histological features suggestive of MTC (**Figure 2**). No lymphatic and vascular invasion was detected, and the surgical margins were free of tumor. The histological examination of the adrenal gland (**Figure 1B**) showed evidence of pheochromocytoma without histological malignant characteristics, specifically necrosis, atypical mitoses, and vascular invasion. Invasion of capsular and peri-adrenal tissues was not detected, and rare tumor cells exhibited intense positive cytoplasmic staining for calcitonin.

After the surgery, hypertension remitted and urinary adrenal hormones reverted to normal levels. However, post-operative serum calcitonin level was high (426 pg/mL) and the stimulation test with calcium gluconate showed increased levels of calcitonin. Subsequent CT and bone scintigraphy with metaIodobenzylguanidine and gallium did not show any conclusive findings. The suspicion of the MEN2A syndrome was confirmed through evaluation of the RET gene. The results showed p.C634R mutation in exon 11 and polymorphisms in p.A432A, p.G691S, and p.S904S.

## Discussion

The patient case can be explained by adrenergic-induced ACS triggered by pheochromocytoma and hyperthyroidism. The delayed diagnosis of pheochromocytoma could be due to the concomitant presence of hyperthyroidism.

Germline RET mutations are generally observed in 98% of patients with MEN2A^22^. In the present case, a mutation was found in the 634 codon of the exon 11 of the RET gene. RET mutations are mostly missense and located in the extracellular domain of RET (exons 10 and 11)^23^ and are correlated with high frequency of pheochromocytoma (more than 50%)^24^. The risk of developing MTC depends on RET mutation and can be prevented with prophylactic thyroidectomy. As such, genetic analysis must be performed in patients with a family history of MEN2A to perform early diagnosis and preventive thyroidectomy and thus avoid cervical neck dissection.

The American Thyroid Association has graded risk development and surgical timing from D (higher risk) to A (lower risk). Mutations at codon 634 of the RET gene correspond to a C classification, which is associated with high risk of aggressive MTC^25^. The phrase is appropriate as it is now stated.

Primary hyperparathyroidism is a frequent complication of MEN2A syndromes^23^. In the present case, this diagnosis was ruled out because of biochemical assessment and surgery results. As parathormon levels were normal pre- and postoperatively, the parathyroid was explored during the surgery, which revealed normal macroscopic anatomy.

In conclusion, ACS was probably secondary to intense coronary vasospasm caused by underlying adrenergic stimulation by pheochromocytoma and hyperthyroidism, which are the first clinical presentations of the MEN2A syndrome.
